# Discovery and cryoEM structure of FPM13, a periplasmic metalloprotein unique to *Francisella*

**DOI:** 10.1371/journal.ppat.1014024

**Published:** 2026-03-27

**Authors:** Daniel L. Clemens, Bai-Yu Lee, Xiaoyu Liu, Z. Hong Zhou, Marcus A. Horwitz

**Affiliations:** 1 Department of Medicine, University of California, Los Angeles (UCLA), Los Angeles, California, United States of America; 2 Department of Microbiology, Immunology and Molecular Genetics, UCLA, Los Angeles, California, United States of America; 3 The California NanoSystems Institute (CNSI), UCLA, Los Angeles, California, United States of America; National Jewish Health, UNITED STATES OF AMERICA

## Abstract

We report the identification and cryoEM structure of the *Francisella* protein FTN_1118, a previously uncharacterized 13 kDa periplasmic protein unique to the *Francisella* genus. The protein was serendipitously discovered during purification of *Francisella* type VI secretion system (T6SS) effector proteins and is hereby designated as FPM13 (*Francisella* Periplasmic Metalloprotein, 13 kDa) based on its cellular and biochemical properties. Identified by the cryoID approach based on our cryoEM density map, FPM13 exists naturally as a cylindrical 18-mer complex with 9-fold dihedral symmetry, formed by stacking two donut-shaped nonamers head-to-head. Measuring ~8 nm in height and outer diameter with a 3.5 nm central channel, the complex features a double-layered wall comprising an inner β-sheet core and an outer α-helical shell. Each FPM13 monomer adopts a compact fold comprising an N-terminus β-strand, an α-helix and two additional β strands at the C-terminus. Inter-ring loop interactions, hydrophobic contacts, and electrostatic interactions between adjacent subunits stabilize the assembly. Biochemical analyses, including APEX-biotinylation and Triton X-114 phase partitioning, confirmed FPM13 as a soluble periplasmic protein. Inductively coupled plasma mass spectrometry (ICP-MS) revealed FPM13 binds iron, copper, and zinc, with alanine substitution of predicted metal-binding cysteine and histidine residues abolishing this capability. Biochemical assays further revealed that wild-type FPM13 catalyzes disulfide bond formation and rescues alkaline phosphatase from reductive inactivation, indicating a role in maintaining periplasmic disulfide bonds. The metal-binding disruption mutant loses this oxidation activity. Deletion of FPM13 in *Francisella novicida* caused no growth defects *in vitro*, in macrophages, or in mice under tested conditions, suggesting functional redundancy may compensate for its absence. This study unveils a novel metalloprotein and demonstrates the power of cryoID in identifying uncharacterized proteins directly from structural data, offering new insights into *Francisella* biology.

## Author summary

*Francisella tularensis*, the cause of tularemia, is a highly infectious bacterium and a Tier 1 U.S. Federal Select Agent with sophisticated survival strategies. While studying *Francisella novicida*, a model organism closely related genetically to the biosafety level 3 agent *Francisella tularensis*, we discovered FPM13, a previously unknown 13 kDa protein unique to this genus. Found unexpectedly during efforts to isolate components of the bacterium’s type VI secretion system, a key virulence factor, FPM13 was visualized using cryogenic electron microscopy and identified via the innovative cryoID method. This revealed a ring-shaped complex of 18 subunits capable of binding metals like iron, copper, and zinc, a function reliant on specific amino acid residues. Our *in vitro* biochemical assays show that FPM13 catalyzes disulfide bond formation. The metal-binding disruption mutant loses this oxidation activity. However, deletion of FPM13 did not impair bacterial growth in lab cultures, immune cells, or animal models, hinting at compensatory mechanisms. This discovery underscores the power of advanced structural biology to uncover hidden microbial components and highlights cryoID as a transformative tool for identifying novel proteins in pathogens.

## Introduction

The *Francisellaceae* species are widely distributed in nature and cause natural infections in a wide range of animals, including mammals, birds, amphibians, fish, mollusks, insects, and protists. Some species are obligate pathogens of animals and humans (e.g., *F. tularensis*), whereas other species are present in the environment and are opportunistic pathogens of humans (*F. novicida* and *F. philomiragia*). Other species of *Francisellaceae* have been associated only with animals (e.g., *F. noatunensis*) or protists (e.g., *F. endociliophora*), and others (e.g., *F. persica*) are endosymbionts that live only in ticks. Tularemia, also known as “rabbit fever”, is a potentially fatal bacterial zoonotic disease whose etiologic agent is *F. tularensis,* a gram-negative coccobacillus and member of the *Francisellaceae* family.

In its natural habitats, *F. tularensis* infects lagomorphs or voles, and blood-sucking insects (e.g., ticks, deer flies, and mosquitoes) serve as intermediate hosts or vectors that can transmit the infection to humans. *F. tularensis* is one of the most infectious bacterial pathogens known [1,2]; inoculation [1] or inhalation [2] of as few as 10 or 25 organisms, respectively, can cause lethal infection in humans. For this reason, and because it has been previously developed as a bioweapon [3], it has been designated as a Tier 1 U.S. Federal Select Agent. Transmission of tularemia can occur from the bite of an infected tick, deerfly or other insects; from handling infected animal carcasses; from eating or drinking contaminated food or water; or from inhaling the bacteria [2].

Macrophages are the primary host cells for *F. tularensis* in animals [4]. After uptake by macrophages via the novel mechanism of looping phagocytosis [5], *F. tularensis* initially resides in a phagosome that resists fusion with lysosomes and fails to acquire acid hydrolases, such as cathepsin D [6]. Within hours, the bacterium escapes from its phagosome and multiplies extensively in the macrophage cytosol [7]. The *Francisella* Pathogenicity Island (FPI), a cluster of ~17 genes, is a major virulence determinant that encodes a Type 6 Secretion System (T6SS) [8]. Disruption of almost any of the FPI genes renders the bacterium unable to escape the phagosome, multiply in host macrophages, and cause disease [9]. Because of this, a major focus of our research has been on purifying and determining the structure and function of the components of the *Francisella* T6SS, especially its secreted effector proteins. Because it shares the same intracellular lifestyle and is genetically very similar to the more pathogenic *F. tularensis*, many of our studies are conducted in the closely related species *F. novicida*, which was first isolated from saltwater taken from Great Salt Lake, Utah [10]. *F. novicida* rarely can cause infections in humans, including an outbreak in a Louisiana prison, with ice machines being identified as the common source [11].

In this study, we determined the cryoEM structure of a native *F. novicida* protein complex serendipitously discovered during T6SS effector protein isolation. Using the cryoID approach, we subsequently identified the cryoEM density map being that of an 18-mer complex of FTN_1118, a previously uncharacterized 13 kDa protein. Based on its periplasmic localization and metal-binding properties identified in this study, we hereby designate it FPM13 (*Francisella* Periplasmic Metalloprotein, 13 kDa). FPM13 is unique to the *Francisella* genus but conserved across species within the genus. In the complex, two donut-shaped nonameric rings stack together in a head-to-head fashion with D2 symmetry. Localization and biochemical assays confirmed that FPM13 is a soluble periplasmic protein capable of binding iron, copper, and zinc. Modeling predicted five residues involved in metal binding. Replacement of these residues abolished metal binding of FPM13. *In vitro* biochemical assays revealed that FPM13 catalyzes disulfide bond formation and that it is able to rescue a model enzyme (alkaline phosphatase, which requires disulfide bonds for enzyme activity) from reductive inactivation. The metal-binding disruption mutant loses this oxidation activity. However, deletion of the protein does not impair bacterial growth *in vitro* or *in vivo* in macrophages or mice, suggesting existence of redundant or alternative pathways that complicate identification of the precise functional role of the metal-binding capability and sulfhydryl oxidation activity of FPM13.

## Results

### Structural determination and identification of the novel *F. novicida* protein FPM13

**Fig 1:**
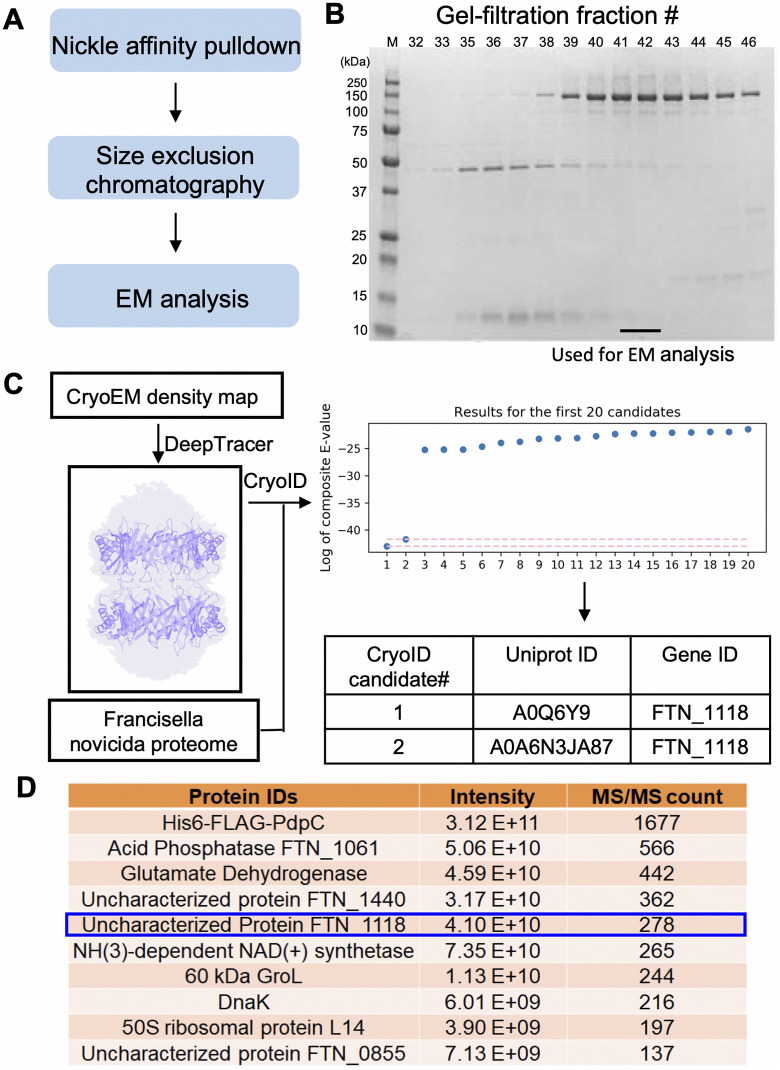
Discovery of FPM13 (FTN_1118) from *Francisella novicida.* **(A)** Workflow for protein sample preparation. **(B)** Coomassie blue stained SDS-PAGE gel for different fractions after gel-filtration. The fraction numbers are labeled. Fractions 41 and 42 are used for EM analysis. **(C)** Workflow for protein identification. The cryoEM map was used to generate a 3D model with predicted amino acid sequence traced by DeepTracer. The traced PDB model and the *Francisella novicida* proteome downloaded from UniProt are subjected to cryoID. The first ranking candidate is FTN_1118 (FPM13). *De novo* model building is performed in Coot. **(D)** The top10 proteins identified by mass spectrometry-based proteomics in the protein sample further verified the protein sample contains FTN_1118 (FPM13).

The novel *F. novicida* protein was discovered in the process of characterizing a 156 kDa *Francisella* T6SS effector protein, PdpC. To determine the structure and function of PdpC, we expressed it with N-terminal FLAG and hexa-His epitope tags in *F. novicida*. The protein was enriched by affinity and size-exclusion chromatography (Fig 1A). A Coomassie stained SDS-PAGE gel showed a dominant band corresponding to a ~ 150 kDa protein, consistent with PdpC (Fig 1B). However, a protein band at about 13 kDa which mostly eluted earlier on gel filtration (indicating a higher molecular weight complex) was also apparent on the Coomassie stained SDS-PAGE gel (Fig 1B). Fractions from gel filtration with the most intense staining for the ~ 150 kDa protein (Fig 1B, Fractions 41 and 42) were analyzed by cryoEM (S1A Fig), with 2-dimensional class averages showing donut-shaped structures (S1B Fig). CryoEM analysis achieved 3.6 Å resolution (S1C-S1E and S2A Figs), and the statistics are summarized in S1 Table. DeepTracer [12] was first used to generate a de novo model. CryoID [13] was subsequently applied, using the *F. novicida* U112 genome as the search database, to perform error-tolerant protein identification, yielding a ranked list of candidate proteins and leading to the identification of FPM13 (FTN_1118) (Fig 1C). Mass spectrometry analysis of the protein sample used for cryoEM analysis confirmed that PdpC is the dominant protein present and also revealed the presence of some other proteins (Fig 1D), with FPM13 being the fifth most abundant protein in the sample used for cryoEM.

**Fig 2:**
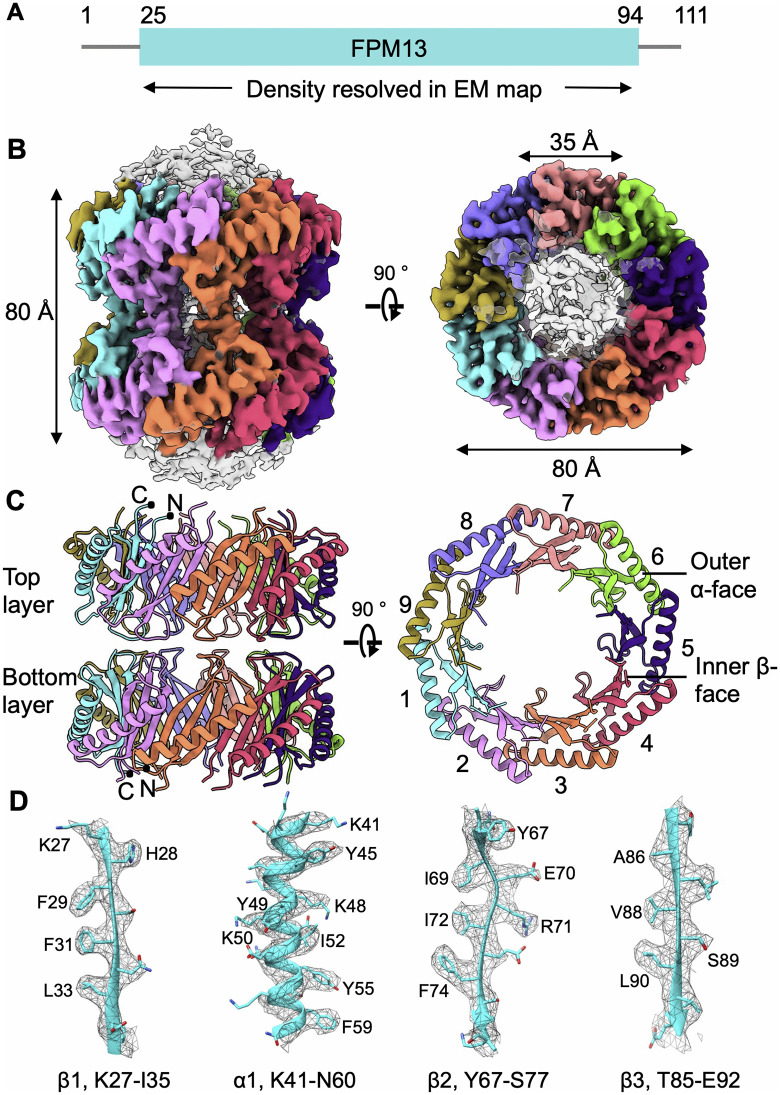
CryoEM structure of FPM13. **(A)** Diagram of *Francisella novicida* (Fn) protein FPM13. The full-length protein consists of 111 amino acids. S25-S94 are resolved in cryoEM map. **(B)** Two different views of the cryoEM density map of FPM13 octadecamer consisting of two nonamer layers stacked tail to tail. The protomers in each nonamer are represented in different colors. Two protomers stacked tail to tail within two layers are in the same color. **(C)** Two different views of FPM13 octadecamer structure. The structure is colored as in **B.** The protomers in one nonamer layer is labeled 1-9. **(D)** Representative density map of α helices and β strands.

**Fig 3:**
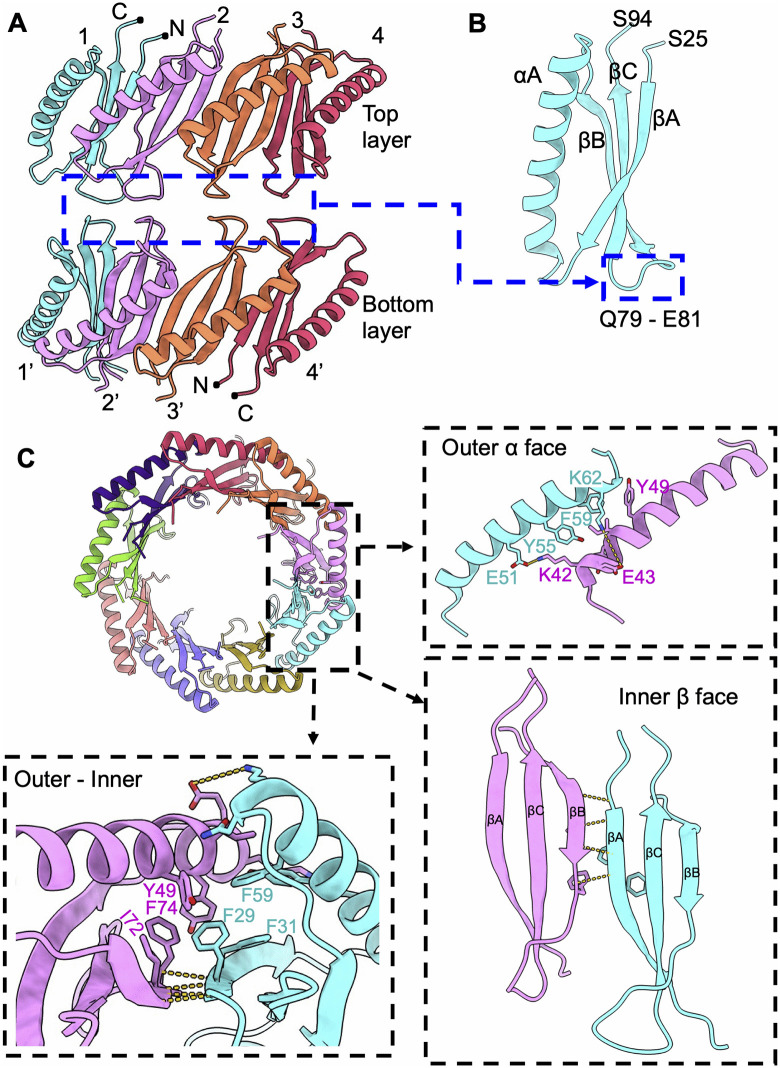
Interactions between FPM13 protomers. **(A)** Front view of the FPM13 assembly; the rear portion is omitted for clarity. **(B)** The loop region mediates the interactions between the top and bottom layers. **(C)** Illustration of the interactions between lateral subunits, with key residues shown in stick representation and labeled.

FPM13 is a protein composed of 111 amino acids. Amino acid residues 25 – 94 were resolved in the cryoEM density map (Fig 2A), with the N- and C-termini (residues 1–24 and 95–111, respectively) being unstructured. The atomic model of the complex (Fig 2B-2D) shows a structure with two layers stacked head-to-head. Each layer has 9 repeating subunits of a monomer (13 kDa), consistent with the sequence of FPM13 and the ~ 13 kDa band seen in the SDS-PAGE gels of the gel filtration samples (Fig 1B). The entire structure has 18 repeats, corresponding to 234 kDa, explaining its high molecular elution profile on gel filtration. The complex measures ~8 nm both in height and in outer diameter, with a 3.5 nm central channel (Fig 2B), within which an uncharacterized density is observed (S2B Fig). It features a double-layered wall with an inner β-sheet core and an outer α-helical shell (Fig 2C). Each monomer adopts a compact fold beginning with a β-strand, followed by an α-helix and two additional β strands (Fig 3A and 3B). Comparison of the atomic model of the FPM13 monomer structure with structures in the Protein Data Bank did not yield any strong matches in a DALI [14] search.

Interactions between FPM13 monomers in the assembly are shown in Fig 3. The top and bottom layers of the 18-mer are stacked primarily through van der Waals interactions, mainly involving the loop regions spanning residues Q79 to E81 of the head-to-head protomers (Fig 3A-3B). Lateral interactions between two subunits are mediated by a combination of hydrophobic (Y49, Y55 and F59) and electrostatic contacts (E51-K42, K62-E43) between the outer α helices (Fig 3C). The inner β sheet is stabilized through backbone hydrogen bonding as well as hydrophobic interactions among side chains (Fig 3C). The outer and inner faces are glued together by hydrophobic residues (Y49, F74, I72, F29, F31 and F59). The interface between two adjacent subunits is substantial, with a buried surface area of 765 Å^2^ at this single interface, suggesting a strong association. We also confirmed the interaction of FPM13 with itself by bacterial 2-hybrid analysis (S3 Fig).

### FPM13 is a soluble periplasmic protein

**Fig 4:**
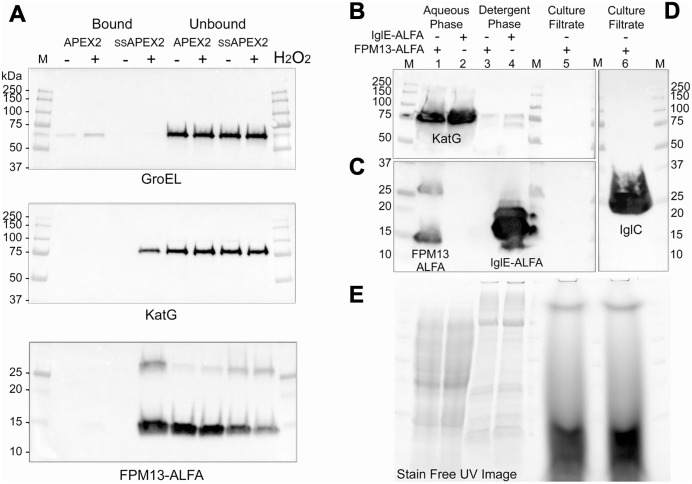
FPM13 is a soluble periplasmic protein. **(A)** FPM13 is biotinylated in the presence of H_2_O_2_ and pulled down by streptavidin-agarose APEX2 that is targeted to the periplasm (ssAPEX2), but not by cytosolic APEX2. GroEL and KatG serve as cytosolic and periplasmic control proteins, respectively. **(B - C)** TX-114 phase extraction shows that FPM13 and KatG are soluble proteins that partition into the aqueous phase, unlike the known lipoprotein, IglE (C, lane 4). FPM13-ALFA is not secreted into the culture filtrate (C, lane5), unlike positive control IglC (**D**, lane 6). Stain free UV-image shows equal loading of lanes being compared **(E).**

Following the structural characterization and identification of FPM13, we next examined its localization within the cell. Bio-informatics analysis of the FPM13 sequence (PsortB [15] and Uniprot [16] annotation) predicted that the first 20 amino acids of the protein preceding a cysteine residue constitute a signal peptide and that the protein is a periplasmic lipoprotein. To verify this prediction, we prepared *F. novicida* expressing FPM13 with a C-terminal ALFA epitope tag (FPM13-ALFA). We found that osmotic based preparations of *F. novicida* periplasmic proteins (e.g., obtained using the Tris-Sucrose-EDTA method) were contaminated with cytosolic proteins, such as GroEL and DnaK, thus complicating their interpretation. To determine more precisely whether FPM13 is a periplasmic protein, we utilized the technique of compartment-specific peroxidase-mediated biotinylation [17] with APEX2, an engineered variant of soybean ascorbate peroxidase [18]. We prepared *F. novicida* that expresses FPM13-ALFA and the engineered ascorbate peroxidase either with (ssAPEX2) or without (APEX2) a signal sequence for secretion (S4 Fig), thereby targeting the ascorbate peroxidase to either the periplasm or the cytoplasm, respectively. As shown in Fig 4A, FPM13-ALFA was biotinylated and pulled down by streptavidin-agarose in the H_2_O_2_ treated *F. novicida* expressing ssAPEX2 targeted to the periplasm, but not in the strain expressing APEX2 lacking a signal sequence or in samples that were not treated with H_2_O_2_. As a positive control, the catalase-peroxidase enzyme, KatG (which has a signal sequence and is predicted to be periplasmic by PsortB) was also pulled down in the H_2_O_2_ treated ssAPEX condition (Fig 4A). In contrast, the cytosolic protein GroEL was not pulled down in the ssAPEX conditions (with or without H_2_O_2_) but was pulled down in the *F. novicida* expressing cytosolically targeted APEX. The ssAPEX2 biotinylation results indicated that FPM13 is located in the periplasm.

To test whether FPM13 is a lipoprotein, as indicated by PsortB, SignalP 6 [19], and Uniprot annotation, we utilized the well-established method of Triton X-114 (TX-114) phase partitioning [20,21]. As a control, we also prepared *F. novicida* expressing C-terminal ALFA-tagged IglE, a component of the T6SS apparatus that is well established as an outer membrane lipoprotein [21,22]. We conducted TX-114 phase separation using the methods described by Brusca and Radolf [20]. As expected, IglE-ALFA partitioned exclusively into the TX-114 phase. In contrast, FPM13-ALFA partitioned exclusively into the soluble phase (Fig 4C), demonstrating a soluble periplasmic protein. As further controls, the periplasmic protein KatG as expected partitioned into the aqueous phase (Fig 4B), while the secreted protein IglC was detected strongly in the culture filtrate (Fig 4D), unlike FPM13-ALFA. Stain free UV image of the gel prior to transblotting confirmed comparable loading of the lanes being compared (Fig 4E). Together, these results establish that FPM13 is a soluble, non-lipoprotein periplasmic protein of *F. novicida.*

### FPM13 is not required for *F. novicida* growth in macrophages *in vitro* or in mice *in vivo*

**Fig 5:**
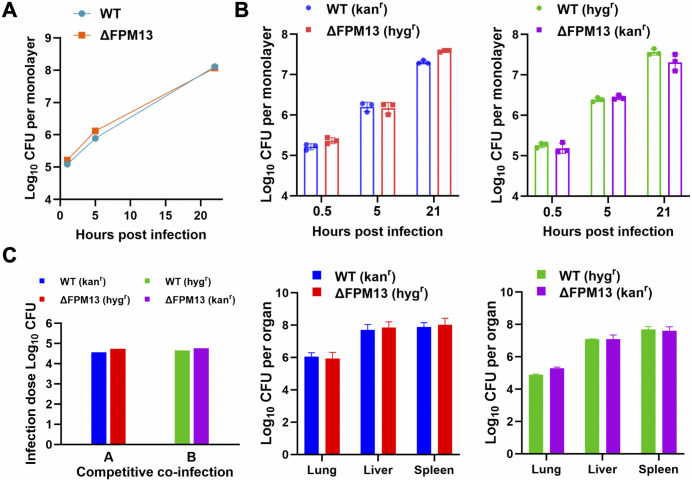
FPM13 is not required for growth in THP-1 macrophages *in vitro* or in mice *in vivo.* **(A)** PMA-differentiated THP-1 macrophages were infected with the wildtype parent (WT) or FnΔFPM13 strain of *F. novicida* and numbers of bacteria in macrophage monolayers determined by plating for CFU at the indicated time points. **(B)** Capacity of *F. novicida* WT and ΔFPM13 strains to compete for intracellular growth in THP-1 macrophages was examined in strains expressing kanamycin (kan) and hygromycin (hyg) resistance markers. Numbers of CFU of each strain in the monolayers was determined at the indicated times by plating monolayer lysates on agar containing kan or hyg. **(C)** Capacity of *F. novicida* WT and ΔFPM13 strains to spread to lung, liver and spleen after intraperitoneal infection was examined in a competition infection experiment using strains expressing hyg or kan resistance. Panel on the left shows the initial CFU dose of each strain used for infection. Middle and right panels show organ burdens of WT and ΔFPM13 strains with the indicated resistance markers.

FPM13 encodes a 111-amino acid polypeptide of unknown function. To determine whether FPM13 plays a role in *Francisella* growth in host cells, we generated a mutant strain of *F. novicida* with deletion of the gene sequence corresponding to amino acid residues 1–92 (FnΔFPM13). PMA-differentiated human THP-1 macrophages were infected with either the wildtype parent or FnΔFPM13 strain, and the bacteria were harvested from the monolayer at different time points after the infection (Fig 5A). At 30 min post-infection, a similar number of wildtype and mutant strains were recovered from the monolayers indicating that there is no distinct difference in uptake of the two strains by host macrophages. After uptake, both the parent and mutant strains exhibited comparable growth kinetics with the bacterial number increasing by ~1 log and 3 logs at 5 h and 22 h post-infection, respectively.

Although FnΔFPM13 showed no obvious growth defect inside of THP-1 macrophages, it was conceivable that the deletion affects its fitness, a trait that often manifests under competitive growth of a mixed wildtype and mutant population in the same host environment. To examine this possibility, we introduced different antibiotic resistance markers (hygromycin and kanamycin) in the wildtype and mutant strains and assayed their ability to outgrow each other *in vitro* in THP-1 macrophages. To correct for any resistance marker associated bias, we performed the macrophage infection experiments with each of the resistance markers on either the wildtype or the deletion strain (Fig 5B). We observed that the wildtype and mutant strains grow equally well in macrophages, with the strain bearing hygromycin resistance showing a slight competitive advantage over the other strain bearing kanamycin resistance.

We next investigated the capability of the wildtype and FnΔFPM13 strains to establish an infection *in vivo* in mice. We pre-mixed the two strains at 1:1 ratio prior to infecting C57BL mice by the intraperitoneal route, and two days later we determined bacterial burdens in the lung, liver and spleen (Fig 5C). The wildtype and FnΔFPM13 strains showed comparable growth in each of the organs suggesting that knocking out FPM13 has no impact on the fitness of the bacterium to spread and multiply in mice.

Taken together, our results show that deletion of FPM13 has no discernible effect on the capacity of *F. novicida* to replicate within macrophages, compete for fitness, or establish infection in mice.

### FPM13 is a metalloprotein

**Fig 6:**
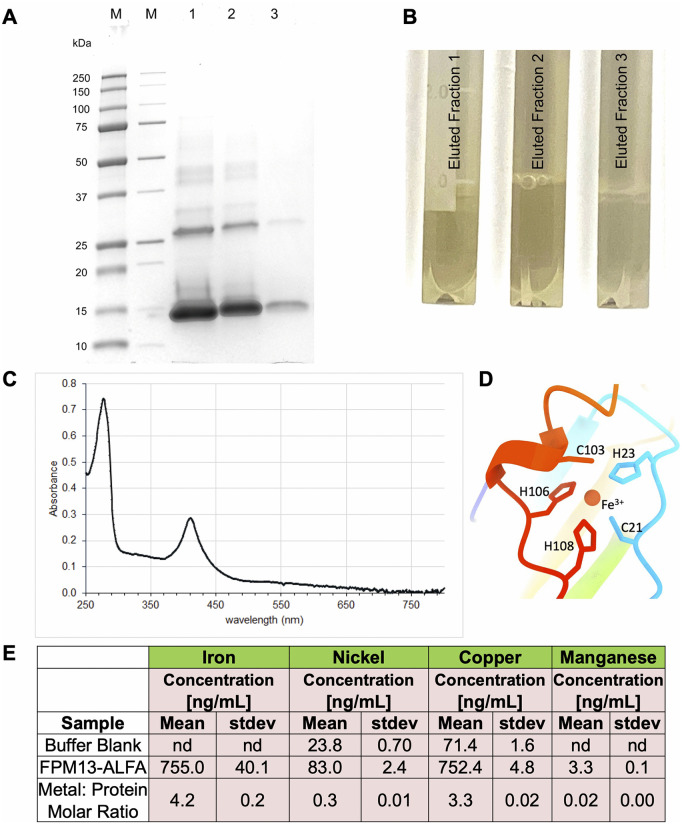
Identification of FPM13 as a metalloprotein. **(A)** Coomassie stained gel showing purification of FPM13-ALFA on ALFA-select resin. Lanes 1 - 3 correspond to consecutive elutions of resin with 0.1 M glycine HCl, pH 2.2. **(B)** Appearance of eluates 1 – 3 demonstrating brown color. **(C)** UV-vis spectrum of fraction #1, showing absorbance peaks at 280 nm and 410 nm. **(D)** Ribbon diagram illustrates possible mechanism of metal binding predicted by Chai-1. **(E)** ICP-MS showing presence of Cu and Fe in the sample eluted from the resin. Data from a buffer blank is also shown. Data represent means of triplicate determinations. “nd” indicates below the detection threshold. Metal to FPM13 octadecamer molar ratios are indicated based on a measurement of 0.87 mg/mL protein in the sample used for ICP-MS.

We noticed that FPM13-ALFA purified by affinity chromatography with anti-ALFA resin was colored, with an absorbance maximum at 410 nm (Fig 6A-6C), suggesting that it is a metalloprotein. Interestingly, FPM13 was originally identified through its binding to Ni-agarose and elution in high molecular weight fractions on gel filtration (Fig 1). The binding to Ni-agarose, and potentially other metals, is explained by the presence of five cysteine and histidine residues in positions well suited for co-ordination of metal ions (Figs 6D and S5). The predicted metal-coordination residues (C21, H23, C103, H106 and H108) are located in an unmodelled region with weak cryoEM density, preventing reliable modeling of either the coordinating residues or a bound metal ion. ICP-MS results confirmed that the protein contains both Fe and Cu in metal:octadecamer molar ratios of 4.2 and 3.3, respectively (Fig 6E).

**Fig 7:**
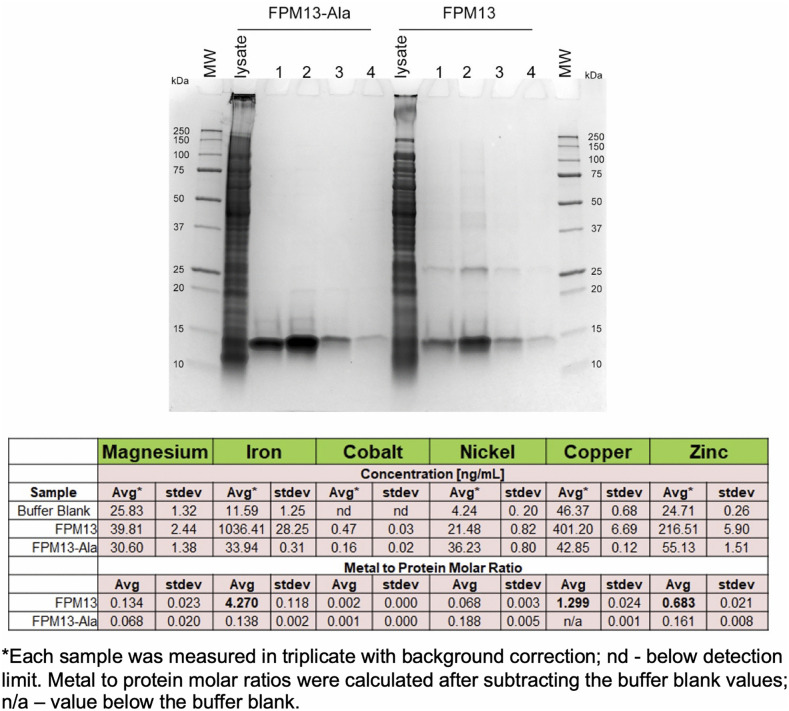
Impact of alanine substitutions on FPM13 metal binding. *F. novicida* strains expressing ALFA-epitope tagged FPM13 or FPM13 with alanine substitutions (FPM13-Ala) were grown in liquid culture, lysed, and the ALFA-tagged proteins were captured by ALFA-select resin and eluted by consecutive applications of acidic buffer with immediate neutralization. The eluted fractions containing the purified FPM13-Ala and FPM13 proteins were pooled and analyzed by ICP-MS. Mg, Co, and Ni content did not differ significantly between FPM13 and FPM13-Ala. Fe, Cu, and Zn content of FPM13 (shown in bold) were significantly greater than that of FPM13-Ala.

To test whether these residues are required for metal binding, we substituted alanine for the five cysteine and histidine residues predicted to coordinate metal ions. The mutant protein exhibited a loss in metal binding (Fig 7). ICP-MS analyses of metals bound by purified parental FPM13 and alanine substitution FPM13 demonstrated binding of iron, copper, and zinc by the parental strain but not the alanine substitution strain. Furthermore, we observed different metal:protein molar ratios in FPM13 prepared under different culture conditions, suggesting that the metals bound may vary depending on environmental conditions.

### FPM13 is not required for acquisition of iron or zinc or for copper detoxification

To evaluate whether FPM13 contributes to metal acquisition or detoxification, we examined the growth of the wildtype, ΔFPM13, complemented, and FPM13-Ala strains in Chamberlain’s chemically defined culture medium (CDM) [23] under defined metal conditions.

When cultured in CDM prepared without iron, all four strains showed equally limited growth. Growth was restored equivalently in all strains as 0.2 – 15 µM iron was added to the medium (S6A Fig). To assess sensitivity to copper, we examined the growth of the four strains in CDM containing the iron and copper chelator bathophenanthrolinedisulfonic acid (BPS, 15 µM), iron (15 µM), and defined concentrations of copper sulfate (0 – 60 µM). All four strains grew comparably in the absence of added copper and showed equal levels of inhibition of growth as the copper concentration was increased from 0.08 – 60 µM (S6B Fig). To evaluate the response of the different strains to zinc, we examined the growth of the 4 strains in CDM containing the zinc chelator N,N,N′,N′-tetrakis(2-pyridinylmethyl)-1,2-ethanediamine (TPEN, 15 µM), iron (15 µM), and defined concentrations of zinc chloride (0 – 15 µM). Because TPEN chelates iron, all four strains showed limited growth in the absence of zinc and grew equally well as zinc was added, which displaced iron from the TPEN (S6C Fig).

To determine whether disruption of FPM13 metal-binding residues affects intracellular growth, we compared the alanine substitution mutant with wildtype, FnΔFPM13, and complemented strains in THP-1 macrophages. No differences in intracellular growth were observed among the strains (S7 Fig).

Taken together, these results indicate that under the metal-limiting and metal-stress conditions tested, deletion or mutation of FPM13 does not affect iron or zinc acquisition, copper detoxification, or intracellular growth.

### FPM13 catalyzes disulfide bond formation

**Fig 8:**
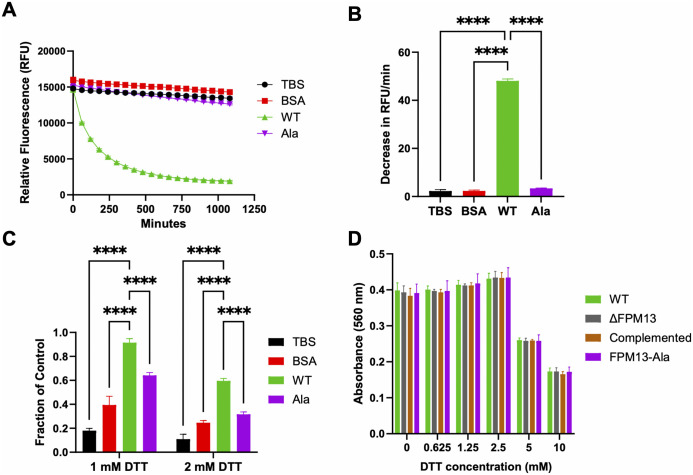
FPM13 exhibits sulfhydryl oxidase activity and rescues alkaline phosphatase from reductive inactivation. **(A)** Fully reduced BODIPY-cysteine (10 µM) was incubated with buffer control (TBS) or with 0.08 mg/mL purified FPM13-ALFA (WT), alanine-substituted FPM13-ALFA (Ala), or BSA control and fluorescence monitored over time (excitation 475 nm, emission 500 – 550 nm). **(B)** Comparison of rate of decrease in BODIPY-cysteine fluorescence per minute over the first 180 minutes. Data shown are the mean ± SEM of 4 replicates. **(C)** Bacterial alkaline phosphatase inactivated with 1 mM or 2 mM DTT, incubated for 1 hour with buffer control (TBS) or with 0.08 mg/mL purified FPM13-ALFA (WT), alanine-substituted FPM13-ALFA (Ala), or BSA control, and alkaline phosphatase enzymatic activity measured. Fraction of alkaline phosphatase activity restored is shown as the mean of 4 independent replicates ± SEM. **(D)** *F. novicida* WT, ΔFPM13, complemented, and FPM13-alanine substituted strains were inoculated into TSBC with 0 – 10 mM DTT at a starting OD of 0.01 and grown for 20 hours at 37°C in 96-well plate format. Data shown are the means ± SEM of 4 replicates. **** indicates p < 0.0001 using ANOVA with Dunnett’s correction for multiple comparisons.

During our further investigation of FPM13 metal-binding properties using BODIPY FL-L-cystine [24], we unexpectedly observed that wild-type FPM13, but not the alanine-substitution mutant, catalyzed the re-oxidation of cysteine to cystine. BODIPY FL-L-cystine consists of two cysteines with BODIPY-labeled amino groups linked via their sulfhydryl side chains. The proximity of the BODIPY fluorophores leads to self-quenching in the oxidized cystine form and an increase in fluorescence occurs with separation of the fluorophores either by reduction or reaction with another sulfhydryl group. To confirm this finding, we fully reduced BODIPY FL-cystine to the maximally fluorescent BODIPY-cysteine with Tris[2-carboxyethyl] phosphine hydrochloride (TCEP)-resin and monitored the time course of re-oxidation after adding TBS buffer control or equal mass amounts of purified wild-type FPM13-ALFA, alanine-substituted FPM13, or bovine serum albumin (BSA). Whereas the wild-type FPM13 rapidly re-oxidized the cysteine to cystine, the alanine substitution mutant, BSA, and TBS control did not (Fig 8A and 8B).

To test whether FPM13 could act on a reduced protein substrate (as opposed to BODIPY-cysteine), we used alkaline phosphatase as a model protein, since alkaline phosphatase requires two disulfide bonds for its structural integrity and enzymatic activity [25]. Treatment of alkaline phosphatase with 1 mM or 2 mM DTT inactivated alkaline phosphatase enzymatic activity, and wild-type FPM13 was more efficient in rescuing alkaline phosphatase from reductive inactivation than equal mass amounts of the alanine-substitution mutant or BSA (Fig 8C), suggesting that FPM13 may play a role in maintaining disulfide bonds in periplasmic proteins. Despite this biochemical activity *in vitro*, no differences in sensitivity to DTT were observed among wild-type Fn, FnΔFPM13, the complemented strain, and a strain in which the FPM13 gene was replaced with a gene corresponding to the alanine-substituted FPM13 (Fig 8D).

These findings demonstrate that FPM13 possesses intrinsic disulfide bond-forming activity. Its loss does not affect DTT sensitivity *in vivo*, likely due to multiple periplasmic oxidoreductases present in *Francisella*, including DsbA1 [26] and FipB [27], that provide redundancy in maintaining the correct disulfide bonding in periplasmic proteins.

### FPM13 homologs are restricted to *Francisella* Clade A

Members in the genus *Francisella* have a diverse geographic distribution and broad host range. Based on a phylogenetic analysis of 425 orthologous single-copy genes, Duron et al. [28] divided *Francisella* into three clades: one corresponding to mammalian pathogens and pathogens in terrestrial environments (including *F. tularensis* and *F. novicida* species); one corresponding to fish pathogens (including *F. philomiragia* and *F. noatunensis*); and a separate distinct clade of tick endosymbionts (including *F. persica*). Kuman et al. [29] performed a pan-genome analysis of 63 *Francisella* genomes and achieved a similar categorization, identifying two distinct clades: Clade A corresponding to *F. tularensis* and *F. novicida* species (species mainly associated with terrestrial animals) and Clade B (*F. philomiragia* and *F. noatunensis*, species associated with aquatic animals). Their analysis also identified a third more diverse cluster, designated Clade C, found mainly in marine environments and comprising *Allofrancisella guangzhouensis* 08HL01032T, *F. frigiditurris* sp. Nov. CA971460, *F. endociliophora* FSC1006, *F. uliginis* sp. nov. TX077310, and *F. halioticida* DSM23729. An additional four outlier species (*F. hispaniensis* FSC454, *F. cf. novicida* 3523, *F. opportunistic asp*. Nov. MA067296, and *F. persica* ATCC VR331) reside between Clades A and B, but closer to Clade A [29].

The FPM13 sequence is unique to *Francisella,* as BLAST [30] homology searches do not identify any homologs outside of *Francisella*. We have found that the FPM13 protein sequence is highly conserved among *Francisellaceae* and homologs are present in all Clade A species of *Francisella* (including *F. holarctica, F. mediasiatica, F. tularensis,* and *F. novicida*) as indicated in S2 Table. However, it is absent from the tick endosymbionts, including *F. persica*, and it is absent from all Clade B species (fish pathogens and marine isolates *F. philomiragia*, *F. saliminara*, and *F. noatuensis*). FPM13 homologs are also absent from almost all Clade C species, including the fish pathogen *F. halioticida*, the ciliate pathogen *F. endociliophora,* and species isolated from water samples such as *A. guangzhouensis* and *F. uliginis*. Intriguingly, an FPM13 homolog is present in the Clade C member *F. frigiditurris*, which was isolated from the water of an air conditioning system.

Our finding that FPM13 is widely present within Clade A strains, many of which are pathogenic to mammals and that it is absent in Clade B strains, which thrive in the marine environment, and many are fish pathogens, implies a potential role of FPM13 in evolutionary adaptation to the Clade A terrestrial environmental niche. Comparing the distribution of FTN_1118 (FPM13 encoding gene) homolog and its neighbor genes in representative genomes among different clades of the genus *Francisella*, we have found that the organization of FTN_1118 homolog and its immediate neighbor genes is highly conserved within Clade A. Gene rearrangement and deletion in this region occur in *Francisella* species outside of Clade A (S8 Fig).

Amino acid sequence of FPM13 and its homologs are highly conserved which suggests a similar structural fold and physiological function. It is also noteworthy that the amino acids involved in metal binding are well conserved in the strains that possess FPM13 homologs, with substitutions being amino acids that are also known to be involved in metal binding (S9 Fig). This conservation suggests a similar structural fold and physiological function.

Further comparisons of *Francisella* species that do and do not possess FPM13 may help to reveal its biological function. For example, it is possible that FPM13 evolved as an adaptation to the reducing environment of hematophagic insect salivary glands and hindgut, an adaptation needed for terrestrial *Francisella* pathogens (except for the tick endosymbionts – which are not transmitted via blood meals) but not for aquatic *Francisella* which do not rely on insect vectors as part of their life cycle.

## Discussion

In this study, we elucidated the cryoEM structure of FPM13, a previously uncharacterized *F. novicida* protein forming a striking 18-mer assembly. Identified directly from cryoEM density maps using the innovative cryoID method, FPM13 was characterized without prior knowledge of its sequence or function. Unlike conventional methods requiring tagging, overexpression, or extensive purification, cryoID enables protein identification in the native state. This approach not only facilitated the discovery of FPM13 but also underscores cryoID’s transformative potential for uncovering novel proteins and macromolecular complexes within their native cellular environments, offering a powerful tool for structural biology.

The discovery of FPM13 as a periplasmic metalloprotein binding iron, copper, and zinc introduces a novel component to *Francisella* biology. Bacterial periplasmic metalloproteins function as transporters that enable the uptake of essential metals such as iron, copper, zinc, and manganese [31], as chaperones that facilitate targeted cofactor insertion into enzymes [32], and as enzymes that support redox modulation to prevent toxicity, thereby maintaining metal homeostasis and contributing to bacterial survival and pathogenicity [33]. These proteins also drive the efflux of excess metals to mitigate cellular damage [34]. In gram-negative bacteria, the periplasm acts as a critical compartment for sensing external metal levels, sequestering ions to protect the cytoplasm, and enabling protein quality control during metal scarcity [35]. For example, in the periplasm of *Escherichia coli*, CueO acts as a copper oxidase metalloprotein that converts harmful Cu(I) to mitigate copper toxicity [36]; ZnuA captures and transports zinc to be released into cytoplasm by ABC trans-membrane transporter [37]; and NDM-1 as a metallo-β-lactamase degraded by periplasmic proteases Prc and DegP at residue-specific sites under zinc limitation, as resolved atomically in live cells [38]. Unlike these, we have not observed any difference between wild-type, FPM13 deletion, or metal-binding disruption mutant strains in sensitivity to copper or in their capacity to grow in chemically defined culture medium with limited iron, copper, or zinc. These results argue against a role for FPM13 in metal homeostasis.

Instead, we discovered that FPM13 catalyzes the oxidation of sulfhydryl groups, suggesting a role in maintaining disulfide bonds in periplasmic proteins and/or in detoxifying volatile sulfhydryl compounds such as those that would be encountered in the guts of insect vectors of terrestrial *Francisella* pathogens. Although most sulfhydryl oxidases are flavoproteins, metal-dependent thiol oxidases, such as the copper-dependent human intestinal enzyme SELENBP1 and nematode SEMO-1, have also been described [39]. This finding highlights a role for FPM13 in periplasmic protein folding and redox homeostasis.

Structurally, FPM13 is distinctive, forming a double-ringed octadecamer not previously observed among periplasmic metalloproteins. The conservation of metal-binding residues, along with the loss of oxidation activity in metal-binding disruption mutant, suggests a structural or mechanistic role for these residues in facilitating sulfhydryl oxidation rather than classical metal transport or detoxification. The structural and functional distinctiveness highlights FPM13 as a novel archetype among periplasmic metalloproteins, warranting further investigation into its mechanistic contributions to *Francisella*’s environmental adaptation.

While deletion of FPM13 did not impair intracellular replication, competitive fitness, or virulence in mice, nor affect growth under metal limitation or reductive stress, these findings likely reflect compensation by functionally redundant pathways. This functional redundancy is not uncommon in bacterial pathogens, where multiple systems ensure robustness, as seen in *Salmonella*’s metal efflux pumps and the multiple periplasmic thiol:disulfide oxido-reductases systems of *E. coli* [40].

Overall, our study unveils the structural and biochemical properties of FPM13, establishing it as a novel metalloprotein with sulfhydryl oxidase activity in *Francisella*. The application of the cryoID approach [13] not only enabled this discovery but also demonstrates its broader potential to identify uncharacterized proteins directly from structural data. These findings advance our understanding of *Francisella*’s molecular repertoire and highlight cryoID as a groundbreaking approach for structural biology and proteomics, with implications for uncovering novel microbial mechanisms.

## Methods

### Ethics statement

All studies described herein were approved by the UCLA Institutional Biosafety Committee (BUA-2021-085-009-A and BUA-2021-101-008-A).

### Generation of *F. novicida* strains

Allelic exchange cassettes for in-frame deletion of FPM13 (ΔFPM) and replacement with a C-terminally ALFA-tagged FPM13 (FPM13-ALFA) including its 1.2 kb genomic upstream and downstream sequences were cloned into pMP590 [41] using standard restriction cloning procedures. The plasmid constructs were confirmed with nucleotide sequencing (Primordium) prior to introducing into *F. novicida* U112. Transformants grown on chocolate GCII agar containing 15 μg/ml kanamycin were spotted on chocolate GCII agar without antibiotics and the presence of the allelic exchange cassette was confirmed using colony PCR. Counterselection using the *sacB* marker on pMP590 was performed by streaking transformants on chocolate GCII agar containing 7% (w/v) sucrose. Strains that grew on CA-sucrose and were sensitive to kanamycin were screened using colony PCR to confirm gene deletion or replacement. A strain expressing FPM13 with alanine substitution of residues C21, H23, C103, H106, and H108 (FPM13-Ala) was generated by a combination of scarless cloning using Type IIS restriction endonuclease Esp3I and standard PCR technique using primers designed for site-specific mutations and suicide vector pJC84 [42] harboring FPM13 and its genomic neighbor sequences as template. Chromosomal deletion and alanine substitutions were validated by PCR amplification of the targeted genomic region and sequencing of the PCR amplicon. Replacement with epitope tagged FPM13 was validated by Western blotting and detecting with ALFA-HRP (Synaptic Systems, 1:4000) antibodies.

APEX2 sequence was amplified by PCR from Vimentin - APEX2 in pECFP (a gift from Alice Ting, Addgene plasmid #66170) [43]. The expression cassette of 3xFLAG-tagged APEX2 with or without the signal peptide sequence from *F. novicida* beta-lactamase (Bla) driven by the promoter of bacterioferritin from *F. tularensis* Live Vaccine Strain was inserted into the *bla* locus in the *F. novicida* strain expressing FPM13-ALFA to replace a genomic region from 30 nucleotides upstream to the first 801 nucleotides of the *bla* gene.

Plasmids pMP607 and pMP633 bearing a kanamycin or hygromycin resistance marker, respectively [41], were transformed into *F. novicida* U112 with and without FPM13 deletion for assaying competitive growth *in vitro* in macrophages and *in vivo* in mice.

### *F. novicida* intracellular growth assay in THP-1 cells

Human monocytic THP-1 cells were maintained in advanced RPMI medium supplemented with 2% heat-inactivated fetal bovine serum (HI-FBS), glutamax, and penicillin/streptomycin. The cells were differentiated with phorbol 12-myristate 13-acetate (PMA) in antibiotic-free medium with 10% HI-FBS for 3 days and infected with overnight cultures of *F. novicida* strains at a ratio of 1:1 (bacterium:cell) at 37°C for 90 min. The infection medium was replaced with medium containing 10 μg/ml gentamicin and incubated at 37°C for 30 min after which the monolayer was washed twice and supplied with fresh culture medium containing 0.2 μg/ml gentamicin. At various time points after infection, monolayers were lysed with 1% saponin in phosphate buffered saline (PBS) at room temperature for 5 min, and the lysates were serial diluted in PBS and spotted on plates for colony-forming units (CFU). For competitive growth assay, equal numbers of *F. novicida* strains bearing different antibiotic resistance markers were premixed prior to infecting PMA-differentiated THP-1 cells. At indicated times, bacteria were harvested from infected monolayers and CFU enumerated after spotting on both agar plates containing kanamycin and agar plates containing hygromycin.

### Competitive growth of *F. novicida* strains in mice

Female C57BL6 mice aged of 7–10 weeks (Jackson Laboratory) were injected intraperitoneally with 0.1 ml normal saline (Cytiva HyClone) containing a mixture of 1x10^5^ CFU each of the *F. novicida* U112 parental and ΔFPM13 strains, carrying kanamycin or hygromycin resistance markers, respectively, or vice versa. Two days later, their lungs, liver, and spleen were harvested and homogenized in PBS. Serial diluted homogenates were plated on GCII chocolate agar containing 10% IsoVitalex and 15 μg/ml of kanamycin or 200 μg/ml hygromycin for enumeration of CFU.

### Bacterial 2-Hybrid protein interaction assay

*F. novicida* gene encoding FPM13 was cloned into each of the four expression vectors (pUT18, pUT18C, pKNT25, and pKT25) of a bacterial adenylate cyclase-based two-hybrid (BACTH) system (Euromedex) in-frame with the coding sequence of two complementary T18 or T25 fragments of *Bordetella pertussis* adenylate cyclase (CyaA). After confirmation by nucleotide sequencing, a pair of compatible plasmids for expression of FPM13 with N- or C-terminal T18 and T25 fusions were co-transformed into the *cya*A negative *E. coli* strain BTH101 by electroporation. Transformants were streaked on Lurie agar containing kanamycin (50 μg/ml), carbenicillin (100 μg/ml), IPTG (1 mM), and X-gal (40 μg/ml), and the plates were incubated at 30°C for two days.

### Protein purification

PdpC: *Francisella novicida* expressing FLAG- and His_6_-epitope tagged PdpC was grown in trypticase soy broth with 0.2% cysteine (TSBC), 5% KCl, 0.1 mg/mL FeSO_4_, and 5 mM betaine to an optical density (540 nm) of 2.0. Bacteria were pelleted by centrifugation (4000 g for 90 min at 4^o^C), resuspended in lysis buffer (50 mM sodium phosphate, pH 7.4, 1 mM EDTA, 1 mM PMSF, 1 mM NEM, and 1:100 protease inhibitor cocktail [HY-K0010, MedChemExpress), and lysozyme (1 mg/mL)] and disrupted by sonication on ice with a probe tip sonicator. The sonicate was clarified by ultracentrifugation (44,400 g for 90 min at 4^o^C) and imidazole, NaCl, and Mega-9 detergent added to achieve concentrations of 10 mM, 0.15 M and 0.2%, respectively. The supernate was rotated overnight with 0.5 mL of high capacity, EDTA compatible, nickel-agarose (Pierce), washed extensively with 50 mM sodium phosphate with 0.15 M NaCl, 1 mM EDTA, 10 mM imidazole, and eluted with an imidazole step gradient (20 mM, 50 mM, 100 mM, 250 mM). Eluted fractions were analyzed by SDS-PAGE and those containing a 150 kDa band corresponding to PdpC were concentrated with a 100 kDa MWCO Centrifugal filter (Amicon Ultra 15), applied to a Sephacryl S200 gel filtration column and eluted in 50 mM sodium phosphate pH 7.4, 0.15 M NaCl, 1 mM EDTA, 0.2% Mega-9. Fractions were analyzed by SDS-PAGE and the fractions corresponding to the peak of the 150 kDa band were concentrated with a 100 kDa MWCO centrifugal filter and used for cryoEM.

FPM13: *F. novicida* expressing FPM13-ALFA was grown in 3 liters of TSBC to an optical density (540 nm) of 2.0. Bacteria were pelleted, resuspended in lysis buffer, sonicated, and clarified by ultracentrifugation as described above. The clarified supernate was added to 0.5 mL of ALFA-select resin CE (Synaptic Systems) and rotated overnight at 4°C. The resin was washed with 20 mM sodium phosphate, pH 8.0, 150 mM NaCl, 0.5% TX-100, followed by washing in 20 mM sodium phosphate, pH 8.0, 150 mM NaCl. The washed resin was transferred to a column and bound protein was eluted with consecutive applications of 0.8 mL of 0.1 M glycine HCl, pH 2.2, 0.8% CHAPS with immediate neutralization by collection of the eluate in tubes containing 0.2 mL of 1 M Tris HCl, pH 8.5. The sample eluted from the column was analyzed by SDS-PAGE, UV-Vis spectroscopy, and inductively Coupled Plasma-Mass Spectrometry (ICP-MS).

### CryoEM sample preparation and image collection

The graphene grids were prepared similarly to as described in the previous work [44] and were used on the same day. An aliquot of 3 μL of purified protein sample was applied to prepared graphene grids. After incubation for 30 s, the grid was blotted for 8 s with 8 blotting force at 4°C and 100% humidity in an FEI Vitrobot Mark IV (Thermo Fisher Scientific). The grid was then flash-frozen in liquid ethane and stored in liquid nitrogen for data collection.

Images were collected using a Titan Krios electron microscope (Thermo Fisher Scientific) at 300 kV. The microscope was operated with the GIF energy-filtering slit width setting to 20 eV in super-resolution mode. Movies were acquired with SerialEM [45] at a magnification of ×81,000, corresponding to a pixel size of 1.1 Å on the sample level, with exposure time of 2 s and total dosage of ~50 electrons/Å2, dose-fractionated into 40 frames. 19,280 movies were collected.

### Single-particle cryoEM reconstruction

The workflow for cryoEM reconstruction is summarized in S2 Fig. Frames of each movie were aligned for correction of beam-induced drift with MotionCor2 [46], generating two averaged images, one with dose weighting (used for particle extraction and further reconstructions) and the other without (for contrast transfer function determination, particles picking and defocus determination). The pixel size of averaged images is 1.1 Å on the specimen scale. The defocus values of images were determined by CTFFIND4 [47]. The following data processing was done with RELION 3.1 [48,49]. 22,936,178 particles were automatically picked, extracted in dimensions of 2 × -binned to 90 × 90 pixels (2.2 Å per pixel) to speed up the data processing procedure. 9,453,347 particles were selected from 2D classification. 3D classification was followed, and 3,040,320 particles were selected. Then the selected particles were re-extracted with a box size dimension of 180 × 180 pixels (1.1 Å per pixel) and subjected to local refinement. The particles after refinement were further re-extracted with a box size dimension of 300 × 300 pixels (1.1 Å per pixel), followed by local refinement. Skip align class3D was applied and two good classes were selected and combined. Another 3D refinement was applied, generating a final 3.6 Å-resolution reconstruction. The resolution of the map was estimated from the gold-standard Fourier shell correlation criterion, FSC = 0.143. Data collection and processing statistics are summarized in S1 Table.

### Protein identification and model building

The reconstructed cryoEM map was used to generate a 3D model trace using DeepTracer [12]. The sequence model provided by DeepTracer was then analyzed with cryoID [13] against the *Francisella novicida* protein database in Uniprot. The top-scoring candidate was identified as FTN_1118 (FPM13). The atomic model of FPM13 was built and manually adjusted in COOT [50]. The model was then refined using Phenix [51] in real space with secondary structure, Ramachandran and rotamer restraints. Refinement statistics of the model were given in S1 Table. Figures were generated with UCSF Chimera [52] and Chimera X [53].

### ICP-MS

ICP-MS analysis was conducted to identify and quantitate metal ions in the samples eluted from the ALFA-resin select CE and buffer controls. The samples were transferred to clean Teflon vessels and digestion was carried out with concentrated HNO_3_ (65–70%, Trace Metal Grade, Fisher Scientific) with a supplement of H_2_O_2_ (30%, Certified ACS, Fisher Scientific) at room temperature for 2 hours. After complete digestion of the sample, it was diluted to a final volume of 5 mL by adding filtered deionized water for analysis. The calibration curve was established using a standard solution while the dwell time was 50 ms with thirty sweeps and three replicates with background correction.

### Mass spectrometry

Protein in the sample used for CryoEM analysis was acetone precipitated by adding 4 volumes of acetone, storing the samples overnight at -20^o^C, and centrifuging at 10,000 g for 10 min at 4^o^C. The pellet was washed twice with 4:1 acetone:water at 4^o^C, air dried for 30 min at room temperature, and stored at -20 °C. Further processing was conducted by the UCLA Proteome Research Center. The pellet was resuspended in 8 M urea, 100 mM Tris-HCl, pH 8.5; reduced with 5 mM tris(2-carboxyethyl)phosphine (TCEP); alkylated with 10 mM iodoacetamide; and digested with sequencing-grade trypsin. The peptide mixture was desalted, fractionated on-line using C18 reversed phase chromatography, and analyzed using tandem mass spectrometry on a Q-Exactive mass spectrometer (Thermo Fisher Scientific). Data analysis was performed using IP2 (Integrated Proteomics Applications) against a Fn U112 database (taxid: 401614) and filtered using a decoy-database estimated false discovery rate of less than 0.01.

### Western blotting

Proteins were separated using Any kD Mini-Protean TGX Stain-free gels (Bio-Rad) and visualized using a ChemiDoc Imaging System (Bio-Rad) prior to transblotting onto a 0.2 μm nitrocellulose membrane. The membrane was blocked with EveryBlock blocking buffer and probed with HRP-conjugated camelid single domain antibody to ALFA epitope tag (Synaptic Systems) or rabbit antibody to KatG or GroEL [54] at a dilution of 1:4000 (anti-ALFA) or 1:1000 (rabbit primary antibodies), respectively. HRP-conjugated goat anti-rabbit antibody (Bio-Rad) was used as a secondary antibody and chemiluminescent signals were developed by incubating with Clarity Western ECL substrates (Bio-Rad) and detected using the ChemiDoc Imaging System.

### APEX2 labeling

The bacteria were grown in TSBC to an optical density of 1.8 and incubated with 1 mM biotin-phenol (Cayman Chemical) for one hour prior to addition of 1 mM H_2_O_2_ (Sigma Chemical Company). Cultures processed identically but without addition of H_2_O_2_ were prepared as controls. After 3 minutes, the *in vivo* biotinylation reaction was terminated by addition of sodium azide, sodium ascorbate, and Trolox to achieve final concentrations of 10 mM, 10 mM, and 5 mM, respectively. The bacteria were pelleted by centrifugation (10,000 g for 10 min) and washed twice with TBS containing 10 mM sodium azide, 5 mM Trolox and 10 mM sodium ascorbate. The washed bacterial pellets were resuspended in RIPA buffer (50 mM Tris HCl, pH 8, 0.15 M NaCl, 1% Nonidet P-40, 0.1% SDS, 0.05% sodium deoxycholate, 1.5 mL for each 7.5 mL of original culture) and sonicated on ice with a probe tip sonicator. Insoluble material was pelleted by centrifugation at 10,000 *g* for 60 min at 4°C and the soluble supernate was incubated overnight with streptavidin-agarose (Novagen, 50 μL of washed resin per 1.5 mL clarified supernate). The resin was washed with RIPA buffer and bound biotinylated proteins were eluted by heating to 90^o^C for ten minutes in SDS-PAGE sample buffer.

### TX-114 phase extraction

*F. novicida* expressing FPM13-ALFA or IglE-ALFA was grown to an optical density of 2.0 in TSBC. PEG-10,000 (10%) was added to the IglE-ALFA culture to induce T6SS expression. Bacteria were pelleted by centrifugation at 10,000 g for 10 min, resuspended in TBS containing 1 mM EDTA, 1 mM PMSF, 1 mM NEM, and 2% TX-114 (2 mg bacterial protein/mL). The culture supernate was saved for analysis of secreted proteins. Suspensions were sonicated on ice with a probe tip sonicator, stirred for one hour in an ice bath, and insoluble material removed by centrifugation at 10,000 g for 1 hour at 4°C. The samples were incubated at 37°C for 10 min to induce phase separation and phases were separated by centrifugation at 20,000 g for 15 min at 23°C. The aqueous (upper) phase was removed and 10% TX-114 added to achieve 2% concentration. Four volumes of TBS containing 0.04% TX-114 were added to the lower detergent phase. The samples were mixed at 4°C for one hour and phase partitioning repeated as before. The aqueous wash and detergent wash of the detergent and aqueous phases, respectively, were discarded and the washing process was repeated twice. Protein of the final washed aqueous and detergent phases and of the culture supernate was precipitated by addition of ten volumes of acetone (-30°C). After 18 hours at -30°C, the precipitated protein was pelleted by centrifugation at 20,000 g for 10 minutes, dried briefly, and resuspended in SDS-PAGE sample buffer for analysis by Western Immunoblotting.

### Measurement of FPM13 cysteine oxidase activity

BODIPY FL L-Cystine (Thermo-Fischer) was dissolved in DMSO at a concentration of 2 mM, diluted to 20 µM with TBS, and fully reduced to maximal fluorescence by overnight incubation (rotating end-over-end) in an argon atmosphere with a 10-fold molar excess of TCEP reducing resin (G Biosciences). The TCEP-resin was removed by centrifugation and 5 µL volumes of the reduced BODIPY-cysteine added to wells of a 384-well black, low volume, round bottom, non-binding assay plate (Corning, #4514). Equal volumes (5 µL) of TBS or 0.08 mg/mL purified FPM13-alfa (WT), alanine-substituted FPM-13 (Ala), or bovine serum albumin (BSA) in TBS were added to the wells and the plate was sealed with MicroAmp Optical Adhesive Film (Thermo Fisher). The decline in fluorescence over time was monitored with a GloMax Explorer (Promega) using an excitation wavelength of 475 nm and emission wavelength of 500 – 550 nm. Data were plotted and analyzed using GraphPad Prism two-way ANOVA with Dunnett’s correction for multiple comparisons.

### Reversal of reductive inactivation of alkaline phosphatase enzymatic activity

Alkaline phosphatase (1 unit/µg/µL; Thermo, Catalog EF0651) was inactivated by 10-fold dilution into 1 mM or 2 mM dithiothreitol (DTT) in TBS and incubation at room temperature for 30 minutes. Full activity control enzyme was diluted 10-fold into TBS without DTT. Aliquots of the DTT-inactivated enzyme were diluted a further 10-fold into TBS or into 0.08 mg/mL purified FPM13-ALFA (WT), alanine-substituted FPM-13 (Ala), or bovine serum albumin (BSA) in TBS and the full-activity enzyme control was diluted 10-fold into TBS. After incubation for 1 hour at room temperature, the alkaline phosphatase activity of the samples was assayed in 96-well plate format by diluting the samples 50-fold with Alkaline Phosphatase Blue Microwell Substrate (Sigma-Aldrich) and measuring absorbance at 595 nm with an iMark microplate reader (BioRad) after 10 minutes of color development (full activity control reaching absorbance of 0.6 – 0.7 absorbance units in comparison with 0.03 absorbance units for TBS control wells). We confirmed that our preparations of BSA and FPM13-ALFA did not have any alkaline phosphatase activity of their own. Data were plotted and analyzed using GraphPad Prism ANOVA with Dunnett’s correction for multiple comparisons.

### Growth of Francisella in TSBC with DTT

*F. novicida* U112 parental, ΔFPM13, complemented, and FPM13-Ala strains were thawed from frozen stocks, grown overnight in Trypticase Soy Broth with 0.2% cysteine (TSBC) to an optical density at 540 nm of ~ 1.8. The strains were diluted to a final optical density (560 nm) of 0.01 with fresh TSBC containing 0 – 10 mM DTT in wells of a 96-well plate, incubated at 37°C, and optical density followed over time with a GloMax Explorer (Promega) plate reader.

### Impact of metals and metal chelators on growth of Francisella in chemically defined medium

*F. novicida* U112 parental, ΔFPM13, complemented, and FPM13-Ala strains were thawed from frozen stocks, grown overnight in Trypticase Soy Broth with 0.2% cysteine (TSBC) to an optical density at 540 nm of ~ 1.8. The bacteria were washed three-times in PBS and grown overnight at 37°C in Chamberlain chemically defined medium [23] prepared without any added iron, copper, or zinc in order to deplete them of residual iron stores. The iron depleted bacteria were inoculated into wells of 96-well plates at a starting optical density (560 nm) of 0.01 in CDM with 0 – 15 µM Fe_2_-EDTA, 0 – 60 µM CuSO_4_, 0 – 15 µM ZnCl_2_, 0 or 15 µM BPS, and 0 or 15 µM TPEN, as indicated in each Figure panel. The plates were grown at 37° C, 5% CO_2_, in a humid atmosphere and optical density (560 nm) was measured after 45 hours in a GloMax Explorer plate reader.

## Supporting information

S1 Fig CryoEM imaging, 2D classification and 3D reconstruction. **(A)** Motion-corrected cryoEM micrograph. The scale bar is 50 nm. **(B)** Representative 2D class averages of FPM13 particles. **(C)** Plot of the FSC as a function of the spatial frequency between the two half maps with indicated resolution at FSC = 0.143. **(D)** FSC curve between the atomic model and the final map with indicated resolution at FSC = 0.5. **(E)** Euler angle distributions of FPM13 particles used for the final reconstruction.
(TIF)

S2 Fig Data processing. **(A)** Workflow of cryoEM data processing. Schematic of classification and refinement procedures used to generate the map obtained in this study (see Methods for details). **(B)** Flexibily attached (grey) or enclosed densities in the FPM13 complex. The density inside is shown in yellow. In the orthogonal review in the right, the peripherally attached (grey in the left) densities are removed to make the yellow density visible.
(TIF)

S3 Fig Bacterial 2-hybrid analysis confirming capacity of FPM13 to interact with itself. *E. coli* BTH101 transformed with a pair of compatible plasmids for expression of FPM13 with N- or C-terminal T18 and T25 fusions were streaked onto LB agar plates containing IPTG and X-gal. While a negative interaction causes no change in color, a positive interaction between the T18 and T25 fusion proteins promotes cAMP production and beta-galactosidase activity in the *E. coli* and turns the color of the bacterial colonies to blue. *E. coli* transformed with plasmids carrying T18-Zip and T25-Zip fusions served as the positive control. *E. coli* transformed with plasmids carrying T25 and T18 without a fusion partner served as the negative control.
(TIF)

S4 Fig Genomic organization and expression of ALFA tagged FPM13 and FLAG tagged APEX2 with or without a signal sequence (SS) for secretion from the *bla* locus of *F. novicida.* **(A)** Gene cassette of FLAG-tagged APEX2 with or without the first 24 amino acids of the *F. novicida* beta-lactamase (Bla) driven by the promoter of *F. tularensis* LVS bacterioferritin (bfr) was inserted into the *bla* locus. The cleavage site of the Bla signal peptide sequence is indicated with a downward arrow. **(B)** Proteins from strains grown in TSBC (lanes 2, 3) or TSBC containing 5% KCl (lanes 4, 5) were separated on SDS-PAGE and the protein profiles imaged by stain free UV imaging (left panel). The proteins were transblotted onto nitrocellulose membrane and probed with FLAG-HRP to detect the expression of APEX2 and ssAPEX2 (middle panel) or with ALFA-HRP to detect the expression of FPM13 (right panel). Lane 1, molecular mass standards.
(TIF)

S5 Fig Why FPM13 (FTN_1118) was enriched in the protein purification. **(A)** Sequence and secondary structure of FPM13 protein. The red arrow indicates the cleavage site between residues A20 and C21, as predicted by SignalP 6.0. **(B)** Chai-1 prediction of FPM13 with Ni^2+^ binding. The ion is shown as a sphere, and key residues are displayed in stick presentation. The binding site is located in the unmodeled top and bottom regions. Key residues involved in ion binding are highlighted in yellow in A.
(TIF)

S6 Fig Impact of iron, copper, and zinc on growth of *F. novicida* strains in chemically defined medium (CDM). **(A)** *F. novicida* wildtype (WT), FPM13 deletion (ΔFPM13), complemented, and alanine-substitution (FPM13-ala) strains were depleted of iron by growth in CDM without added iron and then inoculated at an optical density (560 nm) of 0.01 into CDM with 0 – 15 µM Fe_2_-EDTA and optical density measured after 45 hours at 37°C. (**B**) *F. novicida* strains were processed as described in panel (A) and inoculated into CDM with 15 µM Fe_2_-EDTA, 15 µM BPS (iron/copper chelator) and 0 – 60 µM CuSO4 and absorbance at 560 nm measured after 45 hours at 37°C. **(C)** *F. novicida* strains were processed as described above and inoculated into CDM containing 15 µM Fe_2_-EDTA, 15 µM TPEN (zinc chelator), and 0 – 15 µM ZnCl_2_ and optical density at 560 nm measured after 45 hours at 37°C. In all three panels, the x-axis is shown on a log-scale with “0 µM” metal plotted in the log position of “0.01”. Data shown are the means ± SEM of triplicate determinations.
(TIF)

S7 Fig Intracellular growth assay. Human monocytic THP-1 cells were differentiated with PMA and infected with *F. novicida* bearing FPM13 of the wild-type, in-frame deletion, complementation, or with the metal binding residues substituted with alanine. At the specified time, intracellular bacteria were recovered from the infected THP-1 monolayer, and their numbers determined by counting colony forming units on agar plates after incubation. Data shown are mean and standard deviation of the logarithmic colony forming units (CFU) of six biological replicates.
(TIF)

S8 Fig Distribution of FPM13 and its homologs in *Francisella.* **(A)** Phylogenetic tree of *Francisella* built using Clustal Omega alignment of pilB nucleotide sequences. **(B)** Organization of FPM13 homologs and neighbor genes in *Francisella* genomes. The gray shading indicates interruption of a coding sequence by stop codons or insertion sequences. The strains shown in (A) correspond to those in (B).
(TIF)

S9 Fig Amino acid sequence alignment of FPM13. Colors indicate the physiochemical properties of the amino acids: red (small/hydrophobic), blue (acidic), magenta (basic), and green (hydroxyl/sulfhydryl/amine). Conservation symbols, asterisk(*), colon(:), and period (.) shown below the sequence alignment indicate identical residues, residues of strongly similar properties, and residues of weakly similar properties, respectively. Cysteine and histidine residues predicted to be involved in metal binding are highlighted in yellow. In the strains in which the histidine residue is not conserved, it is replaced by either a tyrosine or asparagine (highlighted in blue), both of which are capable of metal binding.
(TIF)

S1 Table CryoEM data collection, refinement and validation statistics. (DOCX)

S2 Table Sequence-based analysis. *Francisella* (F) strains classified according to phylogenetic analyses of Duron et al., 2018 and Kuman et al., 2020, indicating presence or absence of FPM13 (FTN_1118) homologs.
(DOCX)
