# Supplementary material for: Discovery and cryoEM structure of FPM13, a periplasmic metalloprotein unique to Francisella
Source: PLoS Pathog. 2026 Mar 27;22(3):e1014024. doi: 10.1371/journal.ppat.1014024 (PMC13028475; doi:10.1371/journal.ppat.1014024)
Supplement: S1 Table — (DOCX) [file ppat.1014024.s010.docx]

**S1 Table.** CryoEM data collection, refinement and validation statistics

|  |  | FPM13EMD-70611PDB 9OME |
| --- | --- | --- |
| Data collection and processing |  |  |
| Magnification |  | 81000 |
| Voltage (kV) |  | 300 |
| Electron exposure (e^-^/Å^2^) |  | 50 |
| Defocus range (μm) |  | -1.8 to -2.6 |
| Pixel size (Å) |  | 1.1 |
| Symmetry imposed |  | C1 |
| Particle number |  | 1503607 |
| Map resolution |  | 3.6 |
| FSC threshold |  | 0.143 |
|  |  |  |
| Refinement |  |  |
| Map sharpening *B* factor (Å^2^) |  | -202 |
| Model composition |  |  |
| Non-hydrogen atoms |  | 10602 |
| Protein residues |  | 1260 |
| Ligand |  |  |
| *B* factors (Å^2^) |  |  |
| Protein |  | 56.3 |
| Ligand |  |  |
| R.m.s. deviations |  |  |
| Bond lengths (Å) |  | 0.003 |
| Bond angle (°) |  | 0.645 |
| Validation |  |  |
| MolProbity score |  | 1.50 |
| Clashscore |  | 7.36 |
| Poor rotamers (%) |  | 0.25 |
| Ramachandran plot |  |  |
| Favored (%) |  | 97.55 |
| Allowed (%) |  | 2.45 |
| Disallowed (%) |  | 0 |
