# Supplementary material for: Discovery and cryoEM structure of FPM13, a periplasmic metalloprotein unique to Francisella
Source: PLoS Pathog. 2026 Mar 27;22(3):e1014024. doi: 10.1371/journal.ppat.1014024 (PMC13028475; doi:10.1371/journal.ppat.1014024)
Supplement: S2 Table — Francisella (F) strains classified according to phylogenetic analyses of Duron et al., 2018 and Kuman et al., 2020, indicating presence or absence of FPM13 (FTN_1118) homologs. (DOCX) [file ppat.1014024.s011.docx]

**S2 Table.** Francisella strains classified according to phylogenetic analyses of Duron et al, 2018 and Kuman et al. 2020, indicating presence or absence of FPM13 (FTN_1118) homologs.

| **CLADE** | **Species** | **taxid** | **source** | **Contains FTN_1118 orthologue?** |
| --- | --- | --- | --- | --- |
| **Clade A** | F. novicida U112 | 401614 | water | Yes |
|  | F. tularensis subsp. tularensis SCHU S4 | 177416 | human | Yes |
|  | F. novicida D9876 | 1450527 | human | Yes |
|  | F. tularensis subsp. mediasiatica FSC147 | 441952 | gerbil | Yes |
|  | F. tularensis subsp. holarctica LVS | 376619 | human/vaccine | Yes |
|  |  |  |  |  |
| **Outliers near**  **Clade A** | F. cf. novicida 3523 | 676032 | human | Yes |
|  | F. hispaniensis FSC454 | 1088883 | human | Yes |
|  | F. opportunistica sp. nov. MA067296 | 2016517 | human | Yes |
|  |  |  |  |  |
| **Tick Endo-symbionts** | F. persica ATCC VR331 | 1086726 | tick | No |
|  | F endosymbiont of Dermacentor andersoni | 255914 | tick | No |
|  | F endosymbiont of Haemaphysalis flava | 2007302 | tick | No |
|  | F endosymbiont of Hyalomma asiaticum | 1932426 | tick | No |
|  | F endosymbiont of Amblyomma maculatum | 255919 | tick | No |
|  |  |  |  |  |
| **Clade B** | F. philomiragia ATCC 25015 | 539329 | moribund muskrat | No |
|  | F. philomiragia subsp. philomiragia ATCC25016 | 28110 | water | No |
|  | F noatunensis subsp. noatunensis | 360196 | fresh water | No |
|  | F. noatunensis subsp. orientalis FNO12 | 1390363 | fish (Nile tilapia) | No |
|  | F. salina sp. nov. TX077308 | 573569 | sea water | No |
|  |  |  |  |  |
| **Clade C** | F. endociliophora FSC1006 | 653937 | marine ciliate | No |
|  | A. guangzhouensis 08HL01032T | 594679 | air conditioning system | No |
|  | F. frigiditurris sp. nov. CA971460 | 1542390 | air conditioning system | Yes |
|  | F. halioticida DSM23729 | 549298 | giant abalone | No |
|  | F. uliginis sp. nov. TX077310 | 573570 | seawater | No |
